# Differences in triage category, priority level and hospitalization rate between young-old and old-old patients visiting the emergency department

**DOI:** 10.1186/s12913-018-3257-9

**Published:** 2018-06-15

**Authors:** Sarah Vilpert, Stéfanie Monod, Hélène Jaccard Ruedin, Jürgen Maurer, Lionel Trueb, Bertrand Yersin, Christophe Büla

**Affiliations:** 1 FORS Swiss Centre of Expertise in Social Sciences, University of Lausanne, Géopolis, 1015 Lausanne, Switzerland; 2 Public Health Department of the Canton of Vaud, Av. des Casernes 2, Lausanne, 1014 Switzerland; 3 Réseau Santé Nord Broye, Center for Community Geriatrics, Av. des Sciences 1, 1400 Yverdon-les-Bains, Switzerland; 4 Department of Economics, University of Lausanne, Internef, 1015 Lausanne, Switzerland; 5 Service of Emergency Medicine, University of Lausanne Medical Center (CHUV), Rue du Bugnon 46, 1011 Lausanne, Switzerland; 6 Service of Geriatric Medicine and Geriatric Rehabilitation, University of Lausanne Medical Center (CHUV), Mont-Paisible 16, 1011 Lausanne, Switzerland

**Keywords:** Geriatrics Health services, Emergency department, Hospitalization, Home care impossible

## Abstract

**Background:**

Emergency Department (ED) are challenged by the increasing number of visits made by the heterogeneous population of elderly persons. This study aims to 1) compare chief complaints (triage categories) and level of priority; 2) to investigate their association with hospitalization after an ED visit; 3) to explore factors explaining the difference in hospitalization rates among community-dwelling older adults aged 65–84 vs 85+ years.

**Methods:**

All ED visits of patients age 65 and over that occurred between 2005 and 2010 to the University of Lausanne Medical Center were analyzed. Associations of hospitalization with triage categories and level of priority using regressions were compared between the two age groups. Blinder-Oaxaca decomposition was performed to explore how much age-related differences in prevalence of priority level and triage categories contributed to predicted difference in hospitalization rates across the two age groups.

**Results:**

Among 39′178 ED visits, 8′812 (22.5%) occurred in 85+ patients. This group had fewer high priority and more low priority conditions than the younger group. Older patients were more frequently triaged in “Trauma” (20.9 vs 15.0%) and “Home care impossible” (10.1% vs 4.2%) categories, and were more frequently hospitalized after their ED visit (69.1% vs 58.5%). Differences in prevalence of triage categories between the two age groups explained a quarter (26%) of the total age-related difference in hospitalization rates, whereas priority level did not play a role.

**Conclusions:**

Prevalence of priority level and in triage categories differed across the two age groups but only triage categories contributed moderately to explaining the age-related difference in hospitalization rates after the ED visit. Indeed, most of this difference remained unexplained, suggesting that age itself, besides other unmeasured factors, may play a role in explaining the higher hospitalization rate in patients aged 85+ years.

**Electronic supplementary material:**

The online version of this article (10.1186/s12913-018-3257-9) contains supplementary material, which is available to authorized users.

## Background

Aging of the population challenges health care systems as the number of frail older patients requiring chronic and complex care grows. Emergency departments (EDs) also impacted by this epidemiologic trend as they act as the gateway to medical care for most patients presenting with episodes of acute illness or acute exacerbations of chronic illnesses.

Patients aged 65 years and over account currently for approximately 20 to 30% of all ED visits [1]_,_ a proportion expected to increase in coming years [2–4]. From 2001 to 2009, ED visits by patients aged 65 and older increased by 25% in the United States [2]. This progression was even more pronounced among adults aged 85 years and older, reaching a 30% increase over the same period [2]. Furthermore, adults aged 85 years and over have twice the rate of ED visits as compared with patients in their late 60s [5, 6].

The modes of arrival and referral of elderly patients to the ED, medical diagnoses at ED discharge, as well as discharge dispositions after ED have been widely studied [1, 2, 6–12]. Overall, results show that elderly persons visiting the ED frequently suffer from severe medical conditions [2, 6, 7], generally require more investigations [1, 2, 6–8, 10] and are more often admitted to hospital beds than their younger counterparts [1, 6–8, 10–12]. A few studies also investigated priority levels attributed by triage nurses to elderly patients upon initial ED triage evaluation. They showed that elderly persons are often attributed a high priority level for treatment [1, 6], but also that some older patients might actually be assigned a lower priority level than would have been required [13]. Unfortunately, none of these studies investigated the association between priority level and the risk of hospital admission after the ED visit.

Similarly, whereas there is a large body of literature describing the distribution of triage categories assigned upon ED arrival in older patients [2, 12], little is known on this distribution in the oldest-old patients. In addition, the association between initial triage category and the risk of hospitalization after the ED has usually been assessed retrospectively according to the diagnoses made in the emergency ward [11]. Moreover, these studies did not investigate specifically the oldest-old age group. It would be useful to determine whether associations between triage category and hospitalization risk vary within this specific population among young-old versus old-old patients. Limited consideration has been given so far to the heterogeneity of the elderly population. Indeed, some study showed that the profile of health and function change significantly from age 70 to 85 years, with a steeply rising prevalence of geriatric syndromes and functional decline, as well as an associated increase in health care utilization [14]. Likely, level of priority, triage categories and their associated risk of hospitalization after ED visit may differ in oldest-old compared to young-old patients. Improving the knowledge about triage categories and associated priority levels most prevalent in elderly patients visiting the ED would contribute to improving the understanding of reasons for ED visits in this growing age group. In addition, a better identification of factors associated with hospitalization that differ between young-old and old-old patients visiting the ED is needed to enhance the management of these frail patients.

This study aims to 1) to compare chief complaints (triage categories) and level of priority; 2) to investigate their association with hospitalization after an ED visit; 3) to explore factors explaining the difference in hospitalization rates among community-dwelling older adults aged 65–84 versus 85+ years. More specifically, we first investigated the hypothesis of age-group differences in triage categories, priority levels, and hospitalization rates after an ED visit across the two groups. Then, we examined whether there were age-related specific associations between triage categories and priority levels with hospitalization rates after an ED visit. Finally, we explored how much of the difference in hospitalization rates between the two age groups can be explained by differences in prevalence of patient’s and episode’s characteristics.

## Methods

### Study setting

This study was conducted at the ED of the University of Lausanne Medical Center (CHUV) in Lausanne, Switzerland. The CHUV is a 1′400 beds facility that functions both as a community hospital for the city of Lausanne (~ 300′000 inhabitants) and as a tertiary referral center for Western Switzerland (~ 1.5 million inhabitants).

Patients presenting at CHUV’s ED are first triaged by a trained nurse. Based on patient’s chief complaint or reason for ED visit, this nurse selects the most appropriate corresponding item from the Lausanne Triage and Priority Scale, a 115-items triage scale [15]. Only one item by patient can be selected. This scale includes an extensive list of clinical complaints or events such as contextual factors (intoxication, drug abuse, drowning, etc.), symptoms (thoracic pain, headache, etc.), clinical signs (tachycardia, stridor, deformity, paresis, etc.), as well as miscellaneous items, such as “Request for specific diagnostic test or intervention”, “Home care impossible”, or “Failure to thrive”. These last two categories are proposed for situations, most frequently observed in older persons, where a) unmet needs for in-home care and support lead to hospital admission (“Home care impossible”); b) there are difficulties in triaging in other categories because of a combination of lack of specific complaints and/or symptoms in the context of declining health and function.

For each item, a unique and predetermined level of priority is assigned (e.g. associated priority level of acute chess plaint is Level 1). Level 1 implies immediate treatment (acute situation with immediate vital risk); Level 2 implies medical evaluation and treatment needed within 15 min (urgent situation with no immediate vital risk but at risk of worsening); Level 3 implies treatment needed within 45 min (subacute but stable condition); Level 4 implies assessment needed within 90 min; Level 5 implies assessment needed within 120 min.

When able to walk, patients from Level 5 are usually transferred to the outpatient clinic (about a fifth of the ~ 32′000 adult patients presenting each year to CHUV’s ED), whereas all patients from other levels (~ 25′000) are seen in the ED.

### Data collection

Information about ED visits were collected from two administrative databases recording systematically each patient visiting the hospital or the ED. The two databases were matched through patient and stay identification numbers: 98.1% of all cases were matched across the databases.

Sociodemographic (e.g., age, gender, marital status) as well as patient’s follow-up data (e.g., year of ED visit, discharge disposition after ED) stemmed from the hospital clinical information system, AXYA, that stores patient’s demographics, diagnoses, utilization, payment and disposition status of units’ visits. Data regarding specific information about ED visits, like triage category and priority level, were abstracted from Gyroflux, a software managing patients’ flow in ED. These data are not publicly accessible.

Data from patients visiting the ED were considered for the analysis if the patient: 1) visited the ED between January 1st, 2005 and December 31st, 2009; 2) was 65 years or older; and 3) lived at home (exclusion of patients transferred from nursing homes or other hospitals). Between January 1, 2005 and December 31, 2009, a total of 153′103 ED visits by adult patients (≥ 18 years old) were recorded. ED visits by patients aged 65 years or older (*N* = 45′640) accounted for 29.8% of all ED visits during the period. Among those, 86.8% were made by community-dwelling elderly persons. Finally, individuals (*N* = 438) with incomplete data on all study variables were excluded, leaving a final sample of 39′178 ED visits. During the study period, 44.7% of the sample visited ED ward more than one time, and half of them (23.1%) visited ED three time and more.

### Measures

An ED visit was defined as the period of time the patient stays in the ED area (median length of stay 7h54m). Hospitalization was defined as a patient transfer from ED to inpatient hospital ward (e.g., medical or surgical). Hospitalized patients were opposed to not hospitalized patients that grouped patients who returned home, entered a nursing home or died (0.65%).

For the purpose of the study, the 115-items of the Lausanne Triage and Priority Scale were aggregated into 8 categories, according to a) organ/system-specific conditions (4 categories: cardio-vascular, digestive, respiratory, neurological); b) specific conditions relevant in elderly persons (3 categories: trauma, home care impossible, failure to thrive); c) the “other” category included conditions with small prevalence (urogenital (3.5%), dermatological (2.9%), musculoskeletal (2.5%), ear-nose-throat (2.5%), fever (2.1%), psychiatric problems (0.8%)) and miscellaneous problems (e.g., request for exam, drowning or ingestion of foreign body). Finally, levels of priority were assigned according to the selected item of the Lausanne Triage and Priority Scale.

### Statistical analysis

To examine the age-specific patient’s and episode’s (i.e., priority level and triage category) characteristics, we displayed the distribution frequencies, the 95% confidence intervals, and a Pearson Chi2 statistic of the selected variables for the whole sample (65+ years) and for the two age groups (65–84 years and 85+ years). Additionally, the age-specific associations of sociodemographic variables, priority level, and triage categories with hospitalization after the ED visit were explored, using the ordinary least squares (OLS) regression and taking account of individuals who visited ED several times between 2005 and 2009 by computing a cluster robust standard error for the coefficients. By removing the constant from the OLS regressions, triage categories can be directly interpreted as predicted hospitalization rates. A Chow test [16] was performed which results suggested that OLS regressions should be run separately for the two age groups because of important differences in coefficients between the groups.

Blinder-Oaxaca decomposition methods [17, 18] were used to investigate the difference in hospitalization rates between patients aged 65–84 years and those aged 85+ years. This method is frequently used in economics to analyze, for instance, gender differences in wages, and is also gaining popularity in health disparities research [19].

When applying this method, the difference in predicted hospitalization rates observed in linear regression models for patients aged 65–84 years (group A in the equation below) and patients aged 85+ years (group B) is divided into two components, i.e., (1) the “explained” part, which is attributable to age-related differences in the mean levels of the sociodemographic variables of the model, triage categories and level of priority (predictor variables); and (2) the “unexplained” part, which is attributable to age-related differences in the associations of the predictor variables with hospitalization. Mathematically, the decomposition can be summarized as follows [20]:

$$ \overline{{\widehat{Y}}_A}-{\overline{\widehat{Y}}}_B=\left({\overline{X}}_A-{\overline{X}}_B\ \right){\widehat{\beta}}_A+{\overline{X}}_B\left({\widehat{\beta}}_A-{\widehat{\beta}}_B\right) $$

where group A represents the group of patients aged 65–84 years,

group B represents the group of patients aged 85 years or more,

$\overline{\widehat{Y}}$is the hospitalization rate,

$\left({\overline{X}}_A-{\overline{X}}_B\ \right){\widehat{\beta}}_A$ represents the explained part,

and ${\overline{X}}_B\left({\widehat{\beta}}_A-{\widehat{\beta}}_B\right)$represents the unexplained part.

The Blinder-Oaxaca decomposition is a counterfactual method that simulates how the differences in hospitalization rates between the two age groups would change (i.e., increase or decrease) if patients aged 85+ years had the same characteristics as those aged 65–84 years (“explained” part). Moreover, this method provides the relative contribution of each predictor variable to this explained part. Finally, the unexplained part represents any age-related differences in hospitalization rates that would remain even if patients aged 85+ years had the exact same mean levels of patient’s (i.e., marital status, gender, etc.) and episode’s (i.e., priority level, triage category) characteristics as those aged 65–84 years.

Although our outcome of interest is binary, we chose to apply linear probability models and linear Oaxaca-Blinder decomposition techniques to facilitate the interpretation of the results. Specifically, the use of non-linear binary choice models such as logit or probit models results in additional complications in the context of Blinder-Oaxaca decompositions. Indeed, the explained and unexplained parts are not directly related to the group means of the explanatory variables but need to be expressed in terms of average predicted probabilities, which makes the presentation of results and their interpretation a little more challenging [21]. Yet, to ensure the robustness of our findings, we carefully evaluated potential problems with the use of linear probability models such as the occurrence of potential out-of-bounds predictions (i.e., predicted probabilities below zero or above one). Specifically, the use of linear models results in no predicted probability below zero and less than 1 % of predicted probabilities larger than 1 for each of the two population groups. In addition, we compared the estimation results from our linear probability and linear decomposition techniques with corresponding results based on non-linear logit models. These results are very similar to those based on linear regression techniques (Additional file 1).

As data collected for the study covered a 5-year period, an analysis was first performed to investigate a potential “study year”-related effect. The year of ED visit was initially added as a control variable in OLS regressions and in the Oaxaca-Blinder decomposition. However, as year of ED visits had no significant effect on hospitalization and on the other covariates, data on all study years were finally pooled in our final models.

Data were analyzed using Stata SE v. 13.0. For performing the Blinder-Oaxaca mean decomposition, a Stata command by Jann was used [20].

## Results

### Patient’s and episode’s (i.e., priority level and triage category) characteristics

Characteristics of patients and of ED visits, as well as their comparisons between the two age groups are presented in Table 1. Mean age of the sample population was 78.5 ± 7.9 years (75.4 ± 5.8 years and 89.3 ± 3.4 years in younger and older age groups, respectively). Compared to patients aged 65–84 years, those aged 85+ years were more frequently women (66.3% vs 51.2%) and not married (74.1% vs 50.6%). They also had slightly different levels of priority: a lower proportion of high priority (level 1: 13.8% vs 16.5%) and a higher proportion of low priority (level 4: 15.5% vs 10.9%).

**Table 1:** Characteristics of the elderly population who visited the ED

		65+ years	65–84 years	85+ years	χ2 *P*-value^a^
Sample	N (%)	39,178 (100%)	30,366 (77.5%)	8812 (22.5%)	
Age	Mean (± SD)	78.5 (±7.9)	75.4 (±5.8)	89.3 (±3.4)	
Gender % (95%-CI)	Women	54.6 (54.1–55.1)	51.2 (50.6–51.8)	66.3 (65.3–67.3)	< 0.001
	Men	45.4 (44.9–45.9)	48.8 (48.2–49.4)	33.7 (32.7–34.7)	
Marital status % (95%-CI)	Married	44.1 (43.6–44.6)	49.4 (48.8–49.9)	25.9 (25.0–26.9)	< 0.001
	Not married	55.9 (55.4–56.4)	50.6 (50.1–51.2)	74.1 (73.1–75.0)	
Priority level % (95%-CI)	1 (immediate)	15.9 (15.5–16.3)	16.5 (16.1–16.9)	13.8 (13.1–14.5)	< 0.001
	2 (< 15 min)	28.0 (27.5–28.4)	27.7 (27.2–28.2)	28.9 (27.9–29.8)	
	3 (< 45 min)	23.4 (23.0–23.8)	23.9 (23.4–24.3)	21.9 (21.1–22.8)	
	4 (< 90 min)	11.9 (11.6–12.2)	10.9 (10.5–11.2)	15.5 (14.7–16.2)	
	5 (< 120 min)	20.8 (20.4–21.2)	21.1 (20.6–21.5)	20.0 (19.2–20.8)	
Triage categories (95%-CI)	Cardiovascular	19.6 (19.3–20.0)	19.9 (19.4–20.3)	18.9 (18.1–19.7)	< 0.001
	Trauma	16.3 (16.0–16.7)	15.0 (14.6–15.4)	20.9 (20.1–21.8)	
	Digestive	10.8 (10.5–11.1)	11.6 (11.2–11.9)	8.2 (7.6–8.8)	
	Respiratory	12.7 (12.4–13.0)	12.6 (12.2–12.9)	13.1 (12.4–13.8)	
	Neurological	9.1 (8.8–9.4)	9.4 (9.1–9.7)	8.0 (7.4–8.5)	
	Home care impossible	5.5 (5.3–5.8)	4.2 (4.0–4.4)	10.1 (9.5–10.8)	
	Failure to thrive	4.1 (3.9–4.3)	3.7 (3.5–4.0)	5.4 (5.0–5.9)	
	Other	21.8 (21.4–22.2)	23.7 (23.2–24.2)	15.4 (14.6–16.1)	
Discharge disposition after ED (95%-CI)	Home	37.1 (36.6–37.5)	40.1 (39.5–40.6)	26.6 (25.7–27.5)	< 0.001
	Nursing home	1.4 (1.3–1.5)	0.85 (0.75–0.96)	3.2 (2.9–3.6)	
	Hospital admission	60.9 (60.4–61.4)	58.5 (58.0–59.1)	69.1 (68.1–70.1)	
	Death	0.65 (0.58–0.74)	0.53 (0.45–0.62)	1.1 (0.89–1.3)	

^a^Pearson’s χ2 *P*-value between age group 65–84 and age group 85+

The distribution of triage categories also differed significantly between the two age groups, with 95% confidence intervals not overlapping in all but two (“Cardiovascular” and “Respiratory”) categories. Among patients aged 85+ years, the most frequent triage category was “Trauma” (20.9%) whereas “Cardiovascular” was most prevalent in 65–84 years patients (19.9%). In addition, rates of “Trauma”, “Home care impossible”, and “Failure to thrive” triage categories were higher in the older than in the younger age group. Conversely, rates of “Digestive” and “Neurological” triage categories were lower in patients aged 85+ years than in patients aged 65–84 years.

After the ED visit, 40.1% of patients aged 65–84 years returned home, whereas this proportion was only 26.6% in those aged 85+ years. Thus, patients aged 85+ years were more frequently hospitalized (69.1% vs 58.5%) and transferred to a nursing home (3.2% vs 0.9%) after the ED visit.

### Age-specific associations of hospitalization after an ED visit with patient’s and episode’s (i.E., priority level and triage category) characteristics

Table 2 shows, for each age group separately, results of the OLS regressions that estimated the association of patient’s and episode’s (i.e., priority level and triage category) characteristics with hospitalization after an ED visit. Among patients’ characteristics, gender played a role only in the younger age group (65–84 years) with hospitalization rate 2.1 percentage points higher among men compared to women. In contrast, being married reduced hospitalization rates in both age groups.

**Table 2:** OLS regressions estimating the association of patient’s and episode’s characteristics with hospitalization after an Emergency Department visit in patients aged 65–84 years and 85+ years or more

		65–84 years			85+ years		
		Coefficients (%)	SE (%)	95%-CI	Coefficients (%)	SE (%)	95%-CI
Gender	Women (reference)						
	Men	2.1**	(0.6)	(0.9–3.4)	1.5	(1.2)	(−0.9–3.9)
Marital status	Married (reference)						
	Not married	2.1**	(0.6)	(0.9–3.4)	3.3*	(1.3)	(0.7–5.9)
Level of priority	5 (< 120 min) (reference)						
	4 (< 90 min)	8.9**	(1.3)	(6.3–11.5)	8.3**	(2.8)	(2.9–13.8)
	3 (< 45 min)	1.9	(1.0)	(−0.09–3.9)	0.04	(2.2)	(−4.2–4.3)
	2 (< 15 min)	24.9**	(1.0)	(23.0–26.9)	22.3**	(1.9)	(18.6–26.0)
	1 (immediate)	31.9**	(1.2)	(29.6–34.2)	16.8**	(2.3)	(12.2–21.3)
Triage categories	Cardiovascular	23.9**	(1.3)	(21.4–26.4)	39.8**	(2.6)	(34.8–44.8)
	Trauma	32.7**	(1.0)	(30.7–34.6)	48.5**	(2.1)	(44.4–52.6)
	Digestive	41.4**	(1.3)	(38.8–43.9)	46.8**	(2.8)	(41.3–52.3)
	Respiratory	65.8**	(1.3)	(63.2–68.4)	79.7**	(2.6)	(74.6–84.9)
	Neurological	47.3**	(1.3)	(44.7–50.0)	59.4**	(2.6)	(54.2–64.6)
	Home care impossible	79.6**	(1.7)	(76.3–82.8)	77.8**	(3.2)	(71.4–84.1)
	Failure to thrive	81.2**	(1.3)	(78.8–83.7)	83.9**	(2.0)	(80.0–87.9)
	Other conditions	39.2**	(1.0)	(37.4–41.1)	45.4**	(2.2)	(41.2–49.6)

Dependent variable: hospitalization after an ED visit

SE: Robust standard errors

95% Confidence intervals

** *p*-value < 0.01, * *p*-value < 0.05

Regarding the level of priority, using the lowest priority as reference (i.e., level 5, < 120 min), a general trend was observed with higher priority levels being associated with higher hospitalization rates. This was most evident among the 65–84 age group (except for level 3) whereas the pattern was less linear in the 85+ age group, with the highest and lowest hospitalization rates observed in level 2 and 4, respectively.

Coefficients for triage categories provided in Table 2 can directly be interpreted as the predicted hospitalization rates in a married woman triaged with a priority level 5. All but three triage categories (“Digestive”, “Home care impossible” and “Failure to thrive”) displayed higher predicted hospitalization rated in patients aged 85+ year than in those aged 65–84 years with 95% confidence intervals not overlapping. Interestingly, high predicted hospitalization rates were associated in both age groups with “Home care impossible” and “Failure to thrive”, two non-specific triage categories. Indeed, predicted hospitalization rates of “Home care impossible” and “Failure to thrive” amounted to 77.8 and 83.9%, respectively, among the 85+ age group, and to79.6 and 81.2%, respectively, in the 65–84 age group.

### Age-related difference in hospitalization rates

Table 3 presents the results of the Blinder-Oaxaca decomposition of the difference in hospitalization rates between the two age groups. Given the predictor variables specified in the model, the predicted hospitalization rates after an ED visit was 69.11% in patients aged 85+ years, and 58.53% in patients aged 65–84 years. Thus, the difference in hospitalization rates between the two age groups amounted to 10.58 percentage points.

**Table 3:** Overall Blinder-Oaxaca decomposition of age-related difference in hospitalization rates after an Emergency Department visit

	Estimate (%)	SE (%)
Predicted hospitalization rate for 85+ years	69.11**	(0.54)
Predicted hospitalization rate for 65–84 years	58.53**	(0.33)
Difference in hospitalization rates	10.58**	(0.63)
Total explained difference	3.48**	(0.38)
Total unexplained difference	7.10**	(0.69)
Contribution to explained difference		
Gender	−0.23	(0.18)
Marital status	0.78*	(0.31)
Level of priority	−0.18	(0.18)
Triage categories	2.74**	(0.29)

Dependent variable: hospitalization after an ED visit

SE: Robust standard errors

** *p*-value < 0.01, * *p*-value < 0.05

Age group differences in mean levels of patient’s and episode’s (i.e., priority level and triage category) characteristics explained 33% (3.48/10.58) of the total age-related difference in hospitalization rates after an ED visit (explained difference: 3.48 percentage points). This result means that, everything else being equal, if patients aged 85+ years had the same mean levels in all predictor variables as patients aged 65–84 years, the age-related difference in hospitalization rates would decrease by 3.48 percentage points. The individual contribution of the predictor variables in the explained difference is mainly captured by the age-related differences in mean levels of triage categories (2.74 percentage points).

Finally, 67% of the total difference in hospitalization rates between the two age groups remains unexplained (unexplained difference 7.10 percentage points). The unexplained portion of the difference is attributable to age differences in the associations of the predictor variables with hospitalization and to omitted variables correlated to age.

## Discussion

This study provides unique information on triage categories, priority levels, and hospitalization by age group in a large sample of community-dwelling elderly patients visiting the ED. An important contribution of this study is certainly to show that, even among these older age groups, triage categories and priority levels differed. Indeed, patients from the oldest age group were more frequently triaged in “Trauma” category. “Trauma” was the most prevalent triage category in patients aged 85+ years (accounting for a fifth of all their ED visits). These findings extend those of previous observations that reported trauma-related (e.g., falls and fall-related fractures) rates up to 40% in ED visits by persons aged 65+ years [22] in showing that, even in more advanced age groups, the upward trend continues.

Likewise, this study observed significantly higher rates of “Home care impossible” and “Failure to thrive” in patients aged 85+ years than in their younger counterparts. This observation illustrates the increased risk for oldest-old patients visiting the ED to be triaged with non-specific health conditions and further highlights persisting difficulties in identifying initially specific reasons for ED visit in these patients [13, 23]. Indeed, older patients often present to the ED with unclear history, atypical symptoms and signs and are therefore at higher risk for misdiagnosis than middle-aged adults. In one previous study 51% of older patients initially labeled as “Home care impossible” were eventually diagnosed with significant medical conditions such as infections (24%) and cardiovascular problems (14%) [23]. Another study also observed that, most patients initially labeled as “Failure to thrive” were eventually diagnosed with an acute condition, with more than a third receiving antibiotics [24]. Similarly, mortality in patients labeled as “Social admission” has been found significantly higher than mortality of elderly patients admitted because of specific acute illnesses [25]. Finally, another study showed that 85% of frail elderly patients initially labeled as “Lack of community support” after initial ED examination were eventually identified with significant medical conditions [26].

Overall, results from the present study adds to these observations and further show that non-specific triage categories, such as “Home care impossible” and “Failure to thrive”, may not appropriately reflect underlying conditions that trigger the ED visit and result in high admission rates. In particular, older patients visiting ED with multiple chronic conditions and geriatric syndromes may not fit well these very specific, organ-based triage categories.

Another original contribution of the present study is also to provide unique information on how differences in patients, priority levels, and triage categories observed across the two age groups translated into differences in hospitalization rates after the ED visits. Results show that patients aged 85+ years had consistently higher hospitalization rates than younger patients for all specific triage categories, except “Digestive”. In this regard, new insight is provided by results from the Blinder-Oaxaca decomposition that investigated their respective contribution in explaining the age-related difference in hospitalization rates. Results showed that about a quarter of the predicted difference in hospitalization was explained by the age-related differences in mean levels of triage categories, whereas priority level did not play a role. The remaining unexplained difference is probably related to the interplay between age-related effect of patient’s and episode’s characteristics, and other unobserved age-related factors on hospitalization.

These results strongly suggest that, when presenting at the ED with medical conditions such as cardiovascular or respiratory diseases, oldest-old patients suffered from a more severe form of the disease, but not accounted for by the priority level, than their younger counterparts. Alternatively, physicians might have been more likely to admit these multimorbid patients. Indeed, comorbidities are more frequent with aging and contribute to disease severity as well as mortality as shown inpatients 65 years and over hospitalized for nonmalignant reasons [27] or with advanced heart failure [28].

The oldest-old population is the fastest growing segment of the elderly population, and this growth also impacts ED activities. ED visits and consecutive hospitalization also may have negative outcomes in some elderly patients, especially those reporting difficulties or requiring help to performing their activities of daily living (ADLs) [6]. Two main strategies could contribute to reduce the number of potentially avoidable hospitalization after an ED visit among patients aged 85+ years. First, the use of a specific geriatric assessment tool in addition to the triage tool, could help to attribute more specific triage categories to older patients. This could especially help to better triage those with atypical presentation of acute diseases that are at-risk to being labelled with a catch-all item such as “Home care impossible” or “Failure to thrive” [29]. Second, the development of community care programs for complex older persons with multiple diseases could contribute to prevent acute admissions and bring a valuable alternative to ED visits [6, 30–33], provided that elderly people accept the assistance of healthcare professionals and are compliant with medical care recommendations.

### Limitations

Some limitations should be mentioned. First, results may not be generalizable to other patients admitted to ED in other hospitals where health policies and ED management strategies may differ. In particular the use of a different triage scale, could also modify the percentage and determinants of hospitalization after an ED visit. Nevertheless, the number and proportion of EDs visits [6] and hospitalization rate after an ED visit is in accordance with previous studies [5–8, 10–12, 34].

An additional limitation is related to the use of information from the triage scale, the triage categories, that are not ED diagnoses. Hospitalization risk could differ when using ED diagnoses instead of triage categories as a predictor. However, analyzing triage categories is relevant as the latter reflect the story narrated by the patient or his/her relatives when presenting to the ED. Finally, the use of retrospective ED medical records can be criticized [35, 36]. The present study also has significant strength: it used large and complete sample of computerized systematic medical records. Matched records across database used was high (98.1%) and missing data low. Furthermore, we made no selection on cases, i.e. all ED visits were considered, and avoided thus selection biases. The exhaustiveness of the dataset that included all ED visits of community-dwelling patients aged 65 or older over a prolonged period (from 2005 to 2009) is certainly an additional strength. Finally, the use of the Blinder-Oaxaca decomposition is certainly a nice and original addition to usual analyses.

## Conclusion

This study provides unique information on age differences in patient’s and episode’s (i.e., priority levels and triage category) characteristics among elderly patients visiting the ED, and is the first to explore the specific impact of these differences in explaining the age-related difference in hospitalization rates after an ED visit. Results point to higher rates of non-specific health conditions triage categories in patients aged 85+ years like “Home care impossible” and “Failure to thrive” that were strongly associated with hospitalization after an ED visit. Interestingly, differences in prevalence of triage categories between the two age groups partly explained the age-related difference in hospitalization rates, whereas priority levels did not. Thus, the main part of the age-related difference in hospitalization rates remained unexplained suggesting that age itself remains a major factor associated with hospitalization. These results further support interventions that combine improved care and enhanced social support within as well as upstream the ED to reduce the need for hospitalization after the ED in these vulnerable older patients.

## Additional file

Additional file 1: Appendix 1. Table presenting a comparison of Blinder-Oaxaca decomposition of age-related difference in hospitalization rates after an Emergency Department visit based on OLS regression and logistic regression. (DOCX 13 kb)

## Abbreviations

CHUV: University of Lausanne Medical Center
CI: Confidence interval
ED: Emergency department
OLS: Ordinary least squares
SE: Robust standard errors
