# Supplementary material for: Differences in triage category, priority level and hospitalization rate between young-old and old-old patients visiting the emergency department
Source: BMC Health Serv Res. 2018 Jun 15;18:456. doi: 10.1186/s12913-018-3257-9 (PMC6003168; doi:10.1186/s12913-018-3257-9)
Supplement: Supplementary file 1 — Appendix 1. Table presenting a comparison of Blinder-Oaxaca decomposition of age-related difference in hospitalization rates after an Emergency Department visit based on OLS regression and logistic regression. (DOCX 13 kb) [file 12913_2018_3257_MOESM1_ESM.docx]

Appendix 1

Comparison of Blinder-Oaxaca decomposition of age-related difference in hospitalization rates after an Emergency Department visit based on OLS regression and logistic regression

|  | With OLS regression | | With logistic regression | |  |
| --- | --- | --- | --- | --- | --- |
|  | Estimate (%) | SE (%) | Estimate (%) | SE (%) |  |
| Predicted hospitalization rate for 85+ years | 69.11*** | (0.54) | 69.11*** | (0.54) |  |
| Predicted hospitalization rate for 65-84 years | 58.53*** | (0.33) | 58.53*** | (0.33) |  |
| Difference in hospitalization rates | 10.58*** | (0.63) | 10.58*** | (0.63) |  |
| Total explained difference | 3.48*** | (0.38) | 3.61*** | (0.40) |  |
| Total not explained gap difference | 7.10*** | (0.69) | 6.98*** | (0.69) |  |
| Contribution to explained difference |  |  |  |  |  |
| Gender | -0.23 | (0.18) | -0.24 | (0.19) |  |
| Marital status | 0.78** | (0.31) | 0.80** | (0.32) |  |
| Level of priority | 0.18 | (0.18) | 0.13 | (0.15) |  |
| Triage categories | 2.74*** | (0.29) | 2.91*** | (0.29) |  |

Dependent variable: hospitalization after an ED visit.

SE: Robust standard errors.

** p-value <0.01, * p-value <0.05
